# Cigarette smoke-induced accumulation of lung dendritic cells is interleukin-1α-dependent in mice

**DOI:** 10.1186/1465-9921-13-81

**Published:** 2012-09-19

**Authors:** Fernando M Botelho, Jake K Nikota, Carla MT Bauer, Mathieu C Morissette, Yoichiro Iwakura, Roland Kolbeck, Donna Finch, Alison A Humbles, Martin R Stämpfli

**Affiliations:** 1 Department of Pathology and Molecular Medicine, McMaster Immunology Research Centre, Hamilton, Ontario, Canada; 2 Medical Sciences Graduate Program, McMaster University, Hamilton, Ontario, Canada; 3 Department of Medicine, McMaster University, Hamilton, Ontario, Canada; 4 Center for Experimental Medicine and Systems Biology, Institute of Medical Science, University of Tokyo, Tokyo, Japan; 5 MedImmune LLC, One MedImmune Way, Gaithersburg, MD, USA; 6 MedImmune LTD, Cambridge, UK

**Keywords:** Cigarette smoke, Dendritic cells, T cells, CCL20, Mice

## Abstract

**Background:**

Evidence suggests that dendritic cells accumulate in the lungs of COPD patients and correlate with disease severity. We investigated the importance of IL-1R1 and its ligands IL-1α and β to dendritic cell accumulation and maturation in response to cigarette smoke exposure.

**Methods:**

Mice were exposed to cigarette smoke using a whole body smoke exposure system. IL-1R1-, TLR4-, and IL-1α-deficient mice, as well as anti-IL-1α and anti-IL-1β blocking antibodies were used to study the importance of IL-1R1 and TLR4 to dendritic cell accumulation and activation.

**Results:**

Acute and chronic cigarette smoke exposure led to increased frequency of lung dendritic cells. Accumulation and activation of dendritic cells was IL-1R1/IL-1α dependent, but TLR4- and IL-1β-independent. Corroborating the cellular data, expression of CCL20, a potent dendritic cells chemoattractant, was IL-1R1/IL-1α-dependent. Studies using IL-1R1 bone marrow-chimeric mice revealed the importance of IL-1R1 signaling on lung structural cells for CCL20 expression. Consistent with the importance of dendritic cells in T cell activation, we observed decreased CD4^+^ and CD8^+^ T cell activation in cigarette smoke-exposed IL-1R1-deficient mice.

**Conclusion:**

Our findings convey the importance of IL-1R1/IL-1α to the recruitment and activation of dendritic cells in response to cigarette smoke exposure.

## Background

The adverse effects of cigarette smoke on human health are well established [1,2]. Smoking is the leading cause of chronic obstructive pulmonary disease (COPD), a chronic lung disorders characterized by progressive and largely irreversible airflow limitation [3]. It is widely accepted that chronic inflammation contributes to airflow limitation observed in COPD; macrophages, neutrophils and T lymphocytes are increased in various parts of the lungs [4].

More recently, there is emerging interest in the role of dendritic cells in COPD [5]. Dendritic cells are highly efficient antigen presenting cells and key participants in innate recognition of foreign antigens, fostering activation of adaptive immune responses. Evidence suggests that dendritic cell frequency is increased in the lungs of COPD patients and that expression of maturation markers correlates with worsening of the disease [6]. Dendritic cells have been suggested to contribute to lung tissue damage in smokers through activation of autoreactive T cells and induction of autoantibody responses [7]. In murine models, we and others have shown that cigarette smoke exposure induced the accumulation and maturation of lung dendritic cells [8,9]. Additionally, dendritic cell maturation was associated with the development of emphysema-like lesions [9]. Despite this, mechanisms underlying the recruitment and maturation of pulmonary dendritic cells remain poorly understood.

IL-1R-type1 (IL-1R1) and its cognate ligands, IL-1α and β, play a central role in the initiation of inflammatory processes (reviewed in [10]). IL-1R1 shares homology and mechanisms of intracellular signaling with toll-like-receptors, key sensors of innate pathogen recognition. Studies by Doz et al. demonstrated the importance of IL-1R1, TLR4, and MyD88 (an adaptor signaling molecule shared by IL-1R1 and TLR4) to cigarette smoke-induced inflammation [11]; airway neutrophilia was significantly attenuated in IL-1R1-, TLR4- and MyD88-deficient mouse strains following cigarette smoke exposure. While increased expression of IL-1α and β was observed following cigarette smoke exposure, mechanistic studies revealed that smoke-induced neutrophilic inflammation was IL-1α-dependent, but independent of IL-1β, and relied on crosstalk between hematopoietic and airway structural cells [12]. Studies by Churg et al. further demonstrated that cigarette smoke-induced emphysema formation was, at least in part, IL-1R1-dependent [13].

The objective of this study was to assess the role of IL-1R1 and TLR4 in cigarette smoke-induced accumulation and activation of dendritic cells. We show here that cigarette smoke mediated activation and accumulation of lung dendritic cells was IL-1R1/IL-1α-dependent and independent of IL-1β and TLR4 signaling. IL-1R1-signaling was required in non-hematopoietic lung structural cells for the expression of the dendritic cell chemo-attractant and survival factors, CCL20 and GM-CSF. Finally, CD4^+^ and CD8^+^ T cell activation was IL-1R1-dependent, implicating IL-1-signaling as a mechanism that affects innate and adaptive immune processes.

## Methods

### Animals

BALB/c mice (6–8 weeks old) were purchased from Charles River Laboratories (Montreal, Canada). IL-1R1- and TLR4-deficient mice both on a C57BL/6 background, as well as, C57BL/6 wild type control animals (6–8 weeks old) were purchased from Jackson Laboratories (Bar Harbor, ME, USA). IL-1α-deficient mice on a C57BL/6 background [14] were bred in-house. Mice were maintained under specific pathogen-free conditions in an access-restricted area, on a 12 h light–dark cycle, with food and water provided *ad libitum*. The Animal Research Ethics Board of McMaster University approved all experiments.

### Cigarette smoke exposure protocol

Mice were exposed to cigarette smoke using a whole body smoke exposure system (SIU-48, Promech Lab AB (Vintrie, Sweden)) that was described previously [8]. Mice were exposed to 12 3R4F reference cigarettes with filters removed (Tobacco and Health Research Institute, University of Kentucky, Lexington, KY, USA) for a period of approximately 50 minutes, twice daily, for 4 days (sub-acute exposure), or 5 days/week for 8 weeks (chronic exposure). Control animals were exposed to room air only by removing the cage lid and limiting access to food and water. We previously showed that expression of the stress hormone corticosterone was comparable between cigarette smoke and room air-exposed animals [8].

### Generation of IL-1R1-deficient bone marrow chimeric mice

5 million C57BL/6 wild type or IL-1R1-deficient bone marrow cells were injected intravenously into irradiated (2 doses of 550Rads (11Gray total)) recipient C57BL/6 wild type (WT) or IL-1R1-deficient (knockout (KO)) mice. Recipient mice were on trimethoprim and sulfamethoxazole antibiotic-treated water one week prior to irradiation and two weeks following irradiation. Mice were allowed 8 weeks for reconstitution of hematopoietic bone marrow cells.

### Administration of antibodies

Mice were injected intraperitoneally (i.p.) with 400 μg of anti-IL-1α (clone ALF161; R&D Systems, Burlington, Canada), anti-IL-1β (clone B122; R&D Systems), or Armenian hamster isotype control antibody (Jackson Immunoresearch, Burlington, Canada) 12 hours prior to the first smoke exposure, and then daily 1 hour following the second smoke exposure. Dosing of the antibodies was based on our previous antibody neutralization studies [12,15]. Specificity of the antibodies was confirmed by antibody inhibition assays and reported previously [12].

### Isolation of lung mononuclear cells and flow cytometric analysis

Lung mononuclear cells were isolated as previously described [8]. Briefly, lungs were perfused with 1x phosphate-buffered saline (PBS) and cell suspensions were generated by mechanical mincing and collagenase digestion. Debris were removed by passage through nylon mesh and cells were resuspended in 1x PBS containing 0.3% bovine serum albumin (BSA) (Invitrogen, Burlington, ON, Canada) or in RPMI supplemented with 10% FBS (Sigma-Aldrich, Oakville, ON, Canada), 1% L-glutamine, and 1% penicillin/streptomycin (Invitrogen, Burlington, ON, Canada). 1x10^6^ lung mononuclear cells were washed once with 1x PBS/0.3% BSA and stained with primary antibodies directly conjugated to fluorochromes for 30 minutes at 4°C. 10^5^ live events were acquired on an LSR II (BD Biosciences, San Jose, California) flow cytometer and data analyzed with FlowJo analysis software (TreeStar Inc., Ashland, Oregon). During flow cytometric analysis, side scatter and forward scatter parameters were used to define a live cell gate. All antibodies were purchased from BD Biosciences (San Jose, California) or eBiosciences (San Diego, California) unless otherwise stated. The following antibodies were used for flow cytometric analysis: FITC-conjugated anti-CD11c, PE-Alexa Fluor 610-conjugated CD8, PE-cy7-conjugated anti-CD69, APC-conjugated anti-MHC class II, Alexa Fluor 700-conjugated anti-CD86, APC-cy7-conjugated anti-CD45, and Pacific Blue-conjugated anti-CD3. Qdot605-conjugated anti-CD4 and Qdot655-conjugated anti-B220 were purchased from Invitrogen (Carlsbad, California).

### RNA extraction for fluidigm analysis

RNA was extracted from a single mouse lung lobe. Lung tissues were collected and placed into 200 μl of RNAlater (Qiagen, Mississauga, ON, Canada), stored at −80°C until further use. RNA was extracted using an RNeasy Qiagen kit (Qiagen, Mississauga, ON, Canada). RNA was quantified and normalized, and RNA integrity was assessed by an Agilent Bioanalyzer using the Agilent RNA 6000 Nano Kit (Agilent, Santa Clara, CA, USA). cDNA was prepared using the Super Script III kit from Life Technologies according to the manufacturer’s protocol (Life Technologies, Carlsbard, CA, USA). Relative transcript expression was assessed by Fluidigm Biomark Dynamic array.

### Statistical analysis

Data were analyzed using IBM SPSS Statistics version 18.0 Software (Chicago, Illinois) and expressed as means ± standard errors of the means (SEM). We assessed significance (p < 0.05) using the SPSS Univariate General Linear Model and one- or two-way analysis of variance (ANOVA) was followed by multiple t-tests.

## Results

### Accumulation and activation of lung dendritic cells in response to cigarette smoke exposure is IL-1R1-dependent and TLR4-independent

**Figure 1:**
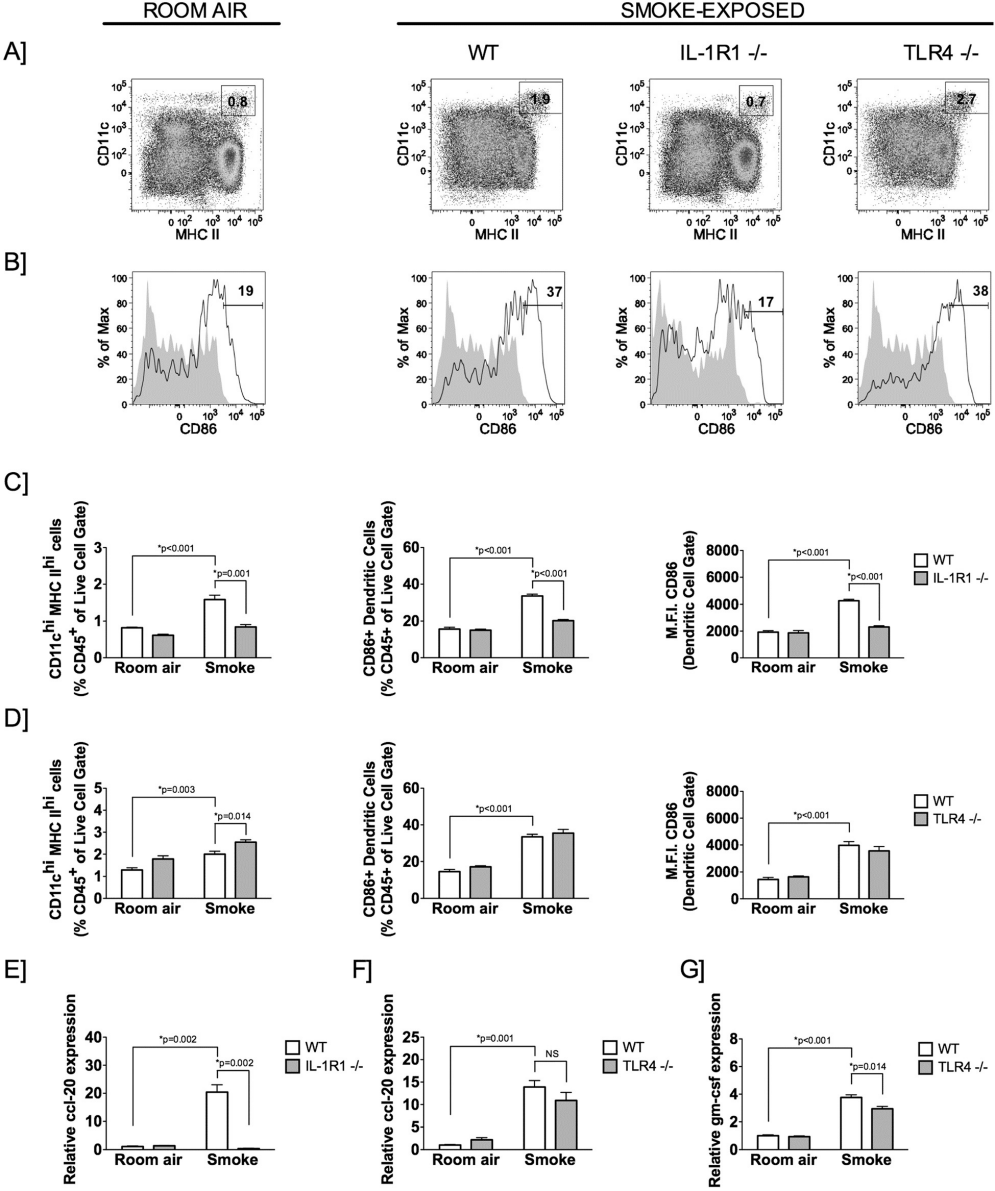
**Cigarette smoke-induced dendritic cell accumulation and maturation is IL-1R1-dependent.** C57BL/6 wild type, IL-1R1-, and TLR4-deficient mice were exposed to cigarette smoke for 4 days. (**A**) CD45^+^ whole lung cells were analyzed by flow cytometry for CD11c^hi^ major histocompatibility class (MHC)-II^hi^ dendritic cells in room air control wild type, and cigarette smoke-exposed wild type, IL-1R1-, and TLR4-deficient mice. (**B**) Flow cytometric analysis of CD86^+^ dendritic cells. Shaded gray histogram represents isotype control stain, while the solid line represents CD86-positive staining. (**C** and **D**) Quantitative analysis of CD45^+^ CD11c^hi^ MHC II^hi^ dendritic cells (left), CD86^+^ dendritic cells (CD11c^hi^ MHC II^hi^) (middle), and mean fluorescence intensity (M.F.I.) of CD86 on dendritic cells (right) of room air and smoke-exposed wild type (WT; white bar), and IL-1R1- or TLR4-deficient (gray bar) mice. Fluidigm analysis of CCL20 mRNA expression in IL-1R1-deficient (**E**) and TLR4-deficient mice (**F**), and GM-CSF mRNA expression in TLR4-deficient mice (**G**). Data presented in A and B are representative of one of five mice from two separate experiments. C to G represent mean ± SEM of at least five mice per group and are representative of at least two independent experiments.

Recent studies by Doz *et al.* have highlighted the importance of IL-1R1 and TLR4 in cigarette smoke-induced lung neutrophilia [11]. Here, we assessed the relative importance of IL-1R1 and TLR4 signaling to dendritic cell accumulation and activation following cigarette smoke exposure. C57BL/6 wild type, IL-1R1-, and TLR4-deficient mice were exposed to room air or cigarette smoke for 4 days. Under these experimental conditions, we previously reported that neutrophilia was significantly attenuated in IL-1R1 deficient animals compared to wild-type controls [12]. Similarly, we observed attenuated neutrophilia in TLR4-deficient mice (data not shown). We observed an increase in CD11c^high^/MHC II^high^ lung myeloid dendritic cells in cigarette smoke-exposed C57BL/6 wild type mice compared to room air controls (Figures 1A). Moreover, smoke exposure led to increased expression of CD86 (B7-2), a dendritic cell activation marker and co-stimulatory molecule (Figure 1B). Lung myeloid dendritic cells and expression of CD86 were significantly decreased in cigarette smoke-exposed IL-1R1-deficient mice compared to wild type controls (Figure 1A and C). In contrast, cigarette smoke exposure led to accumulation of myeloid dendritic cells and increased expression of CD86 in TLR4-deficient mice (Figure 1A and D). These findings suggest an accumulation and activation of myeloid dendritic cells in response to cigarette smoke exposure that is IL-1R1-dependent and redundant of TLR4. 

As previously reported in BALB/c mice, we observed an increase in CD11c^+^/B220^+^ plasmacytoid dendritic cells in C57BL/6 mice following cigarette smoke exposure (Table 1) [15]. A similar increase in plasmacytoid dendritic cells was observed between cigarette smoke-exposed C57BL/6 wild type and IL-1R1 deficient mice (Table 1). Collectively, these data suggest that cigarette smoke-induced accumulation and activation of lung myeloid but not plasmacytoid dendritic cells is IL-1R1-dependent and TLR4-independent. 

**Table 1:** **Frequency of B220**^**+**^**CD11c**^**+**^**plasmacytoid dendritic cells in cigarette smoke-exposed mice**

	**Room air [%]**	**Cigarette smoke [%]**	**P value**
WT	0.070 ± 0.014	0.200 ± 0.001	<0.0001
IL-1R1KO	0.080 ± 0.012	0.220 ± 0.044	0.0001
TLR4KO	0.325 ± 0.126	0.360 ± 0.054	0.5891

Four-day room air and cigarette smoke-exposed C57BL/6 wild type (WT), IL-1R1-knockout (KO) and/or TLR4KO mouse whole lung tissue were analyzed, by flow cytometry, for the percentage of B220^+^/CD11c^+^ plasmacytoid dendritic cells. Data are presented as mean +/− standard deviation (SD).

### Expression of dendritic cell chemotactic and survival factors is IL-1R1-dependent

Next, we investigated whether IL-1R1 signaling was required for the expression of dendritic cell chemoattractant and survival factors. We focused our analysis on CCL-20 based on previous reports that CCR6, the receptor for CCL20, is critically required for dendritic cell accumulation in response to cigarette smoke [9]. Consistent with changes observed in lung dendritic cell frequency, robust up-regulation of CCL20 was observed in wild-type mice, while CCL20 expression was not increased in IL-1R1-deficient mice (Figure 1E). In agreement with the cellular data, we observed a similar induction of CCL20 in cigarette smoke-exposed TLR4-deficient and wild type mice (Figure 1F). As previously reported, cigarette smoke exposure was associated with a significant increase in GM-CSF expression (Figure 1G) [15], a known dendritic cell survival and maturation factor [16]. We observed a modest decrease in GM-CSF expression in TLR4-deficient compared to wild type mice, contrasting our previous observations that cigarette smoke-induced expression of GM-CSF was abrogated in IL-1R1 knock-out mice [15]. These data suggest that IL-1R1, but not TLR4 is critically required for the expression of key dendritic cell survival and chemotactic factors in response to cigarette smoke exposure.

### Smoke-induced dendritic cell accumulation is IL-1α dependent

**Figure 2:**
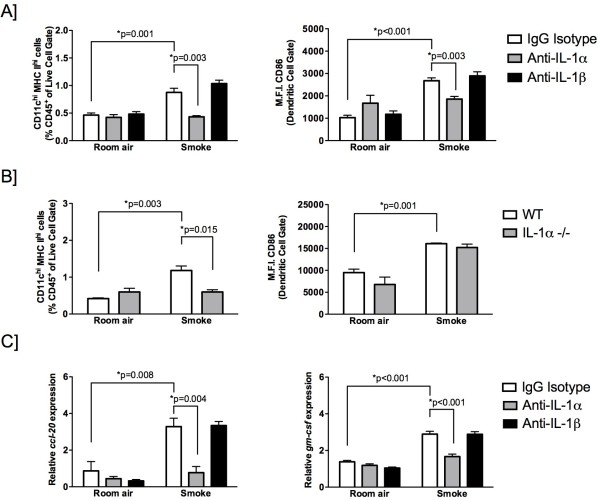
**Cigarette smoke-induced increases in lung dendritic cells, CCL20 and GM-CSF expression is IL-1α-dependent.** (**A**) BALB/c were exposed to cigarette smoke for 4 days. Hamster IgG isotype control (white bar), anti-IL-1α (gray bar), or anti-IL-1β (black bar) antibody were injected intraperitoneally daily starting one day prior to room air or smoke exposure. Data show flow cytometric analysis of CD11c^hi^ MHC II^hi^ dendritic cells (left) and expression of CD86 (right) (M.F.I., mean fluorescence intensity). (**B**) Dendritic cell frequency and expression of CD86 was examined in smoke-exposed IL-1α-deficient (gray bar) and wild type (white bar) mice. (**C**) Expression of CCL20 (left) and GM-CSF (right) in 4-day smoke-exposed mice injected with hamster isotype control, anti-IL-1α or anti-IL-1β antibodies. Data are presented as the mean ± SEM of at least five mice per group and are representative of at least two independent experiments.

IL-1 exists as two isoforms, IL-1α and IL-1β, both of which signal through the IL-1R1. We previously reported increased expression of both ligands in response to cigarette smoke exposure [12]. We next examined whether accumulation and activation of dendritic cells in response to cigarette smoke exposure was IL-1α and/or IL-1β-dependent. BALB/c mice were exposed to cigarette smoke for 4 days. Anti-IL-1α or anti-IL-1β blocking antibody, or an isotype control antibody were delivered daily to cigarette smoke-exposed mice. Anti-IL-1α, but not anti-IL-1β, significantly attenuated dendritic cell frequency and maturation (as assessed by CD86 expression) (Figure 2A). Similarly, we previously reported that cigarette smoke-induced neutrophilia was IL-1α, but not IL-1β-dependent [12]. 

To corroborate the observation that dendritic cell accumulation was IL-1α-dependent, we exposed wild type C57BL/6 and IL-1α-deficient mice to room air or cigarette smoke for 4 days. Consistent with the antibody neutralization studies, IL-1α was critically required for dendritic cell accumulation in response to cigarette smoke exposure (Figure 2B). Of note, CD86 expression was not altered in the absence of IL-1α (Figure 2B). In agreement with the cellular data, we observed attenuated expression of CCL20 and GM-CSF in cigarette smoke-exposed mice treated with anti-IL-1α, but not anti-IL-1β antibodies (Figure 2C). Thus, the IL-1R1 ligand IL-1α, but not IL-1β, is necessary for cigarette smoke-induced dendritic cell accumulation in the mouse lung.

### Dendritic cell accumulation following chronic smoke exposure is IL-1R1/IL-1α dependent

**Figure 3:**
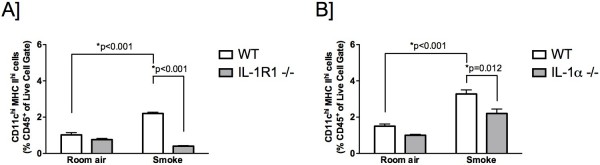
**Increases in lung dendritic cells following chronic cigarette smoke exposure is IL-1R1/IL-1α-dependent*.*** C57BL/6 wild type, IL-1R1- (**A**), and IL-1α-deficient (**B**) mice were exposed to cigarette smoke for 8 weeks. Whole lung cells were analyzed for CD45^+^ CD11c^hi^ MHC II^hi^ dendritic cells by flow cytometry. Results are presented as mean ± SEM of at least five mice per group. Data shown are representative of at least two independent experiments.

The studies pursued thus far focused on dendritic cell accumulation and maturation in response to subchronic cigarette smoke exposure. We next investigated whether dendritic cell accumulation observed following chronic cigarette smoke exposure was similarly IL-1R1/IL-1α dependent. To this end, C57BL/6 wild type, IL-1R1-, and IL-1α-deficient mice were exposed to cigarette smoke for 8 weeks. As seen in Figure 3A, chronic cigarette smoke exposure induced a significant increase in the frequency of lung dendritic cells. The magnitude of dendritic cell accumulation was comparable between acute and chronic cigarette smoke exposure. In accordance with our findings after acute cigarette smoke exposure, IL-1R1-, and IL-1α-deficiency attenuated the accumulation of lung dendritic cells (Figure 3A and B).

### IL-1R1 expression is necessary on non-haematopoeitic structural cells for expression of CCL20 and GM-CSF

**Figure 4:**
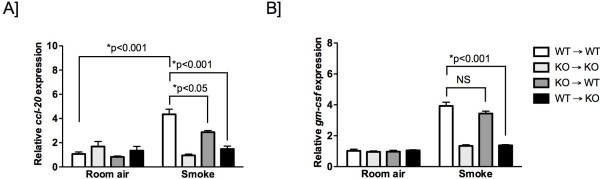
**Expression of IL-1R1 on non-haematopoetic cells is necessary for smoke-induced expression of CCL20 and GM-CSF*.*** Chimeric mice were generated as follows, WT to WT: C57BL/6 wild type bone marrow transferred into irradiated C57BL/6 wild type mice; KO to KO: IL-1R1-deficient bone marrow transferred into irradiated IL-1R1-deficient mice; KO to WT: IL-1R1-deficient bone marrow transferred into irradiated C57BL/6 wild type mice; WT to KO: C57BL/6 wild type bone marrow transferred into irradiated IL-1R1-deficient mice. Chimeric mice were exposed to cigarette smoke for 4 days. Expression of CCL20 and GM-CSF mRNA was assessed by fluidigm analysis. Results are presented as mean ± SEM of at least five mice per group. Data are representative of at least two independent experiments.

Next, we assessed the importance of IL-1R1 expression on haematopoeitic and non-haematopoetic cells for the production of CCL20 and GM-CSF. This study was based on our previous observations that crosstalk between IL-1α + hematopoietic cells and the IL-1R1+ epithelial cells regulates smoke-induced neutrophilia [12]. Significantly increased expression of CCL20 and GM-CSF was observed in cigarette smoke-exposed mice following transfer of C57BL/6 wild type bone marrow (WT) into irradiated (to deplete haematopoetic cells) wild type mice (WT → WT) (positive control) (Figure 4A and B). No increase in CCL20 and GM-CSF expression was observed in irradiated IL-1R1-deficient recipients reconstituted with IL-1R1-deficient bone marrow (KO) into (KO → KO) (negative control), as well as, IL-1R1-deficient mice that were reconstituted with bone marrow from C57BL/6 wild type mice (WT → KO). Bone marrow chimeric mice generated by the transfer of IL-1R1-deficient bone marrow into C57BL/6 wild type irradiated recipient mice (KO → WT) showed increased expression of GM-CSF and CCL20 in response to cigarette smoke exposure. While GM-CSF expression in KO → WT was comparable to WT → WT chimeric mice, we observed a partial reduction in CCL20 expression. Taken together, our findings suggest that expression of IL-1R1 on non-haematopoeitic structural cells is necessary for cigarette smoke-induced expression of CCL20 and GM-CSF. 

### Cigarette smoke-induced expansion of activated T cells is IL-1R1/ IL-1α dependent

**Figure 5:**
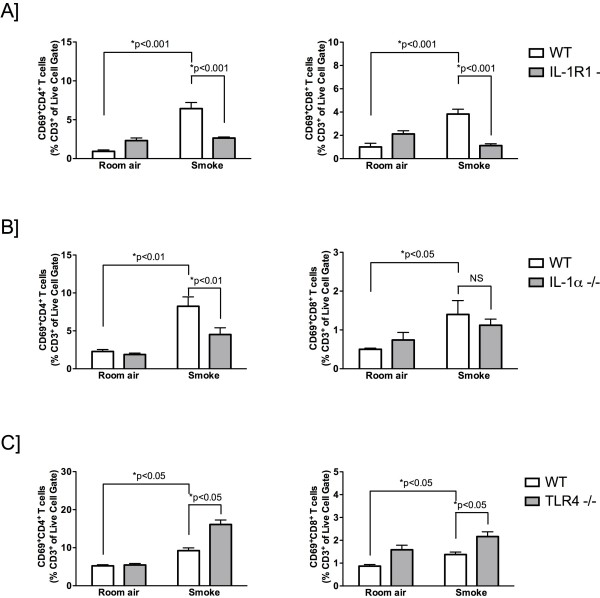
**Cigarette smoke-induced activation of CD4 T cells is IL-1R1/IL-1α-dependent.** C57BL/6 wild type control and IL-1R1- (**A**), IL-1α- (**B**) and TLR4-deficient mice (**C**) were exposed to room air or cigarette smoke for 4 days. Data show flow cytometric analysis of lung CD69^+^ CD4^+^ T cells (CD3^+^ cells) (left panels) and CD69^+^ CD8^+^ T cells (right panels). Results are presented as mean ± SEM of at least five mice per group. Data are representative of at least two independent experiments.

Dendritic cells are essential for initiating potent T cell responses [17] and we previously observed T cell activation following exposure to cigarette smoke [8]. We therefore assessed whether IL-1R1 and IL-1α are required for the expansion of activated T cells in response to cigarette smoke exposure. C57BL/6 wild type, IL-1R1-, and IL-1α-deficient mice were exposed to cigarette smoke for 4 days. Cigarette smoke exposure significantly increased the frequency of lung CD4 and CD8 T cells expressing the activation marker CD69 (Figure 5). Expansion of activated CD4 T cells was attenuated in cigarette smoke-exposed IL-1R1- and IL-1α-deficient mice. Of note, attenuation of CD8 T cells activation was only observed in IL-1R1-deficient mice, while IL-1α deficiency did not impact CD8 T cell activation. Interestingly, in contrast to IL-1R1- and IL-1α-deficient mice, smoke-exposed TLR4-deficient mice showed an increased frequency of CD69+ CD4+ and CD8+ T cells. Thus, consistent with decreased dendritic cell frequency and maturation, activation of CD4 T cells is IL-1R1-dependent. 

## Discussion

Several investigations of mechanisms mediating cigarette smoke-induced inflammation have implicated IL-1R1/TLR-4 signaling [11,13], a canonical inflammatory signaling pathway central to innate immune processes [10]. In the present study, we investigate the influence of these signaling pathways on dendritic cell accumulation in response to cigarette smoke.

COPD is characterized by a complex immunopathology involving innate and adaptive immune cells [3,18]. Neutrophils and macrophages are thought to be the major participants at early stages of the disease, releasing damaging proteolytic enzymes, while emerging evidence suggest that autoimmune processes may contribute to sustained inflammation at later stages of the disease [19]. Although mechanisms mediating autoimmune processes in COPD remain unclear, dendritic cells are of notable interest. Dendritic cells are pivotal in the induction of adaptive immune responses and link innate and adaptive immunity [20]. Of note, dendritic cells accumulate in the lungs of COPD patients, and we and others have shown lung dendritic cells to expand and become activated in murine models of cigarette smoke exposure [8,9].

Utilizing IL-1R1- and TLR4-deficient mice, we show that smoke-induced accumulation and activation of lung dendritic cells is IL-1R1-dependent, but redundant of TLR4 signaling. In contrast, we observed a similar increase in the proportion of lung plasmacytoid dendritic cells in wild type and IL-1R1 deficient mice in response to cigarette smoke-exposure. This finding is in agreement with our previous observations that intervention with an anti-GM-CSF antibody did not affect accumulation of plasmacytoid dendritic cells [15]. While cigarette smoke has been shown to contain biologically relevant levels of the TLR4 ligand LPS [21], our findings demonstrate that LPS contamination does not contribute to dendritic cell accumulation in the lungs. In agreement with our observations, Maes *et al.* reported TLR4-independent dendritic cell expansion and maturation after prolonged smoke exposure, although a reduction was observed at earlier time points (5 weeks) [22]. Mechanisms that contributed to this transient TLR4 dependency were not addressed by Maes *et al.*[22]. Differences between our studies may relate to the genetic background of the mice, as variation in susceptibility of mouse strains to cigarette smoke has previously been reported [23,24]. Alternatively, differences in smoke exposure systems utilized between the two studies may account for the observed disparity in the TLR4 dependency.

Cigarette smoke exposure is associated with increased expression of IL-1α and IL-1β [12]. Both cytokines signal through IL-1R1 [10], and may contribute to dendritic cell accumulation in response to cigarette smoke. Anti-IL-1α antibodies and IL-1α-deficiency, but not anti-IL-1β antibodies, reduced smoke-induced dendritic cell accumulation. Despite decreased accumulation of dendritic cells, we observed increased expression of the activation marker CD86 in IL-1α-deficient mice. This finding contrasts our observations using anti-IL-1α antibodies; administration of anti-IL-1α antibodies attenuated both accumulation and activation of dendritic cells. Differences in phenotype between the gene knockout studies and antibody intervention may be explained by the strain’s adaptation to the IL-1α deficiency. Nevertheless, we demonstrate for the first time an IL-1α-dependent and IL-1β-independent mechanism by which cigarette smoke regulates dendritic cell expansion. Similarly, we recently reported that smoke-driven neutrophilic inflammation is IL-1α/IL-1R1-dependent and redundant of IL-1β [12]. Our findings contrast observations by Churg *et al.* reporting that smoke-induced neutrophilia was IL-1β dependent [13]. This study was pursued using pharmacological inhibitors of caspase-1 to block processing and maturation of bioactive IL-1β and a single acute exposure to whole smoke from 3 cigarettes. Hence, differences in the experimental approach may account for the differential requirement for IL-1α and IL-1β. Moreover, Churg *et al.* did not assess the impact of caspase-1 inhibitors on dendritic cell expansion, precluding a direct comparison to our observations. Given that both IL-1α and IL-1β are expressed in COPD and during episodes of acute exacerbation [12], therapies targeted at the receptor rather than the individual ligands may be more relevant for limiting inflammation and exacerbations in COPD.

Mechanisms that contribute to IL-1α expression in response to cigarette smoke exposure are the subject of active investigation. It is currently not understood whether components of cigarette smoke directly activate IL-1α expression or whether danger associated molecular patterns (DAMPs) that are released secondary to cigarette smoke-induced cell death lead to IL-1α expression; as there is compelling evidence that cigarette smoke exposure induces both apoptotic and necrotic cell death [25-27]. For example, HMGB-1, a DAMP molecule, has been shown to be elevated in COPD airways [28], and may induce along with other DAMPs IL-1α expression. Alternatively, IL-1alpha may be directly released from dying cells and serve as an alarmin. Further analysis of these pathways is warranted, as these mechanisms may provide rationale for the design of novel pharmacotherapies.

CCL20 is one of the most potent dendritic cell chemoattractants [29]. CCL20 expression is increased in the airways of COPD patients [30], and expression of its receptor, CCR6, is critical for the recruitment of dendritic cells to the lungs of smoke-exposed mice [9]. Mirroring the accumulation of dendritic cells, CCL20 expression was IL-1R1-dependent and TLR4-independent. Mechanistically, we show using bone marrow chimeric mice that IL-1R1 expression on structural cells was required for smoke-induced CCL20 expression. Given that hematopoietic cells predominantly express IL-1α in mice exposed to cigarette smoke [12], our findings suggest that crosstalk between IL-1α + hematopoietic cells and the IL-1R1+ epithelial cells underlies CCL20 expression and dendritic cell recruitment.

In addition to recruitment, increased expression of dendritic cell survival factors may also contribute to dendritic cell expansion in response to cigarette smoke exposure. We previously reported that GM-CSF, a dendritic cell survival and maturation factor, is upregulated following cigarette smoke exposure and that administration of GM-CSF ligand or receptor neutralizing antibodies attenuated smoke-induced lung dendritic cell expansion [15]. Hence, attenuated GM-CSF expression in IL-1R1-deficient mice may contribute to decreased dendritic cell survival. However, TLR4-deficient mice, in which we did not observe attenuated dendritic cell accumulation, showed a modest, yet statistically significant, reduction in GM-CSF expression. These findings suggest that GM-CSF-mediated dendritic cell survival may only partially account for dendritic cell accumulation.

Dendritic cells efficiently present antigens and express costimulatory molecules that engage and activate T cells and, therefore, orchestrate the development of productive adaptive immune responses [17]. Both IL-1 and TLR4 signaling have been implicated in regulating dendritic cell function, and consequently, shaping T cell-mediated immunity [10]. Of note, components of cigarette smoke have been demonstrated to have suppressive and/or activating activity on immune cells [31,32]. For instance, nicotine in cigarette smoke has been shown to stimulate dendritic cell function, yet, suppress T cell activation [32,33]. More recently, we and others have shown that cigarette smoke activates dendritic cells and T cells in the mouse lung. Consistent with our dendritic cell findings, we show smoke-driven CD4^+^ T cell activation to be IL-1α- and IL-1R1-dependent and TLR4-independent. Attenuation of CD8^+^ T cell activation was only observed in IL-1R1-, but not in IL-1alpha deficient mice. This may point towards a differential regulation of CD4^+^ and CD8^+^ T cell activation in response to cigarette smoke exposure. Future experimentation, however, is required to solidify this interpretation. Interestingly, we observed increased CD4^+^ and CD8^+^ T cell activation in TLR4-deficient mice in response to cigarette smoke, consistent with the finding that TLR4 can function as a negative regulator in lung inflammatory processes [34]. Thus, we propose that IL-1α-dependent and TLR4-independent smoke-driven dendritic cell expansion and maturation mediates subsequent activation of lung resident T cell. The latter is of particular interest as emerging literature suggest that inflammatory processes mediated by T cells may persists for years after smoking cessation [35,36]. While the specificity of this T cell response remains to be elucidated, there is active discussion whether persistent activation of dendritic cells triggers autoimmune processes in smokers, contributing to the pathogenesis of COPD [19].

## Conclusions

Our study reveals the significance of IL-1α and its receptor, IL-1R1, to cigarette smoke-induced accumulation and maturation of lung dendritic cells. We show this process to be independent of TLR4-signaling and IL-1β-mediated mechanisms. Taken together, these data support the targeting of IL-1α/IL-1R1 and dendritic cell function as a strategy for managing smoke-induced inflammation and processes underlying the pathogenesis of COPD.

## Competing interests

MRS holds funding support from MedImmune. RK and AAH are employees of MedImmune LLC, Gaithersburg, MD; DF is an employee of MedImmune LTD, Cambridge, UK. All of the other authors declare that they have no competing interests.

## Authors’ contributions

FMB was responsible for conceptualization of mouse experiments, experimentation, data analysis, and preparation of the manuscript. JKN provided support for mouse experimentation, discussion, and manuscript preparation. CMTB and MCM provided support for mouse experimentation and discussion. YI provided access to IL-1α-deficient mice and contributed to the discussion of the data. RK, DF, and AAH assisted in conceptualization of experiments, discussion of data, and provided feedback for the manuscript. MRS supervised the project and played an instrumental part in conceptualizing experiments and the preparation of the manuscript. All authors read and approved the final manuscript.
